# Drug treatment for oral submucous fibrosis: an update

**DOI:** 10.1186/s12903-023-03488-9

**Published:** 2023-10-12

**Authors:** Xueru Chen, Hui Xie, Jincai Guo

**Affiliations:** 1 Department of Pharmacy, Changsha Stomatological Hospital, Changsha, 410006 China; 2 School of Stomatology, Hunan University of Chinese Medicine, Changsha, 410006 China; 3 School of Pharmacy, Hunan University of Chinese Medicine, Changsha, 410208 China; 4 Changsha Stomatological Hospital, No. 389 Youyi road, Tianxin district Changsha, Hunan, China

**Keywords:** Oral submucous fibrosis, Drug treatment, Clinical trials

## Abstract

**Objective:**

The aim of this review is to evaluate the different medicinal interventions available for the management of oral submucous fibrosis (OSF).

**Materials and methods:**

We conducted a comprehensive electronic search on PubMed, Web of Science, and Cochrane Library databases for articles related to OSF patients treated with medications from December 2011 to September 2022. GRADE system was used to evaluate the evidence quality. The reporting of the systematic review is in accordance with the Preferred Reporting Items for Systematic Reviews and Meta-Analyses (PRISMA) protocol. The main outcomes were the improvement of maximum mouth opening, burning sensation, cheek flexibility, and tongue protrusion.

**Results:**

Twenty-nine randomized controlled trials (RCTs), five clinical trials (CCTs) were included, and the use of drugs for OSF treatment were evaluated. Drugs like steroids, hyaluronidase, pentoxifylline, lycopene, curcumin, dpirulina, aloe vera, omega3, oxitard, allicin, colchicine have been used. It was found that drugs with evidence high quality were salvia miltiorrhiza combined with triamcinolone acetonide, lycopene, pentoxifylline, curcumin, and aloe vera, and those with evidence moderate quality were allicin, colchicine, omega 3, and oxitard.

**Conclusion:**

Based on the results of our comprehensive analysis, for long-term treatment, we found lycopene with low side effects, whereas for relieving the symptoms of severe burning sensation, aloe vera is the most effective. Although the recent review has made some progress, drug therapy for OSF remains unclear, and more high-quality RCTs are needed to identify better treatments for OSF.

**Supplementary Information:**

The online version contains supplementary material available at 10.1186/s12903-023-03488-9.

## Introduction

Oral submucous fibrosis (OSF) is an insidious and chronic oral mucosal disease. It is a potentially malignant disorder of the oral mucosa with a malignant transformation rate of 4.2% [1]. The initial manifestation of OSF is inflammation, followed by loss of blood vessels and fibrosis visible, and blanching of the oral mucosa with a marble-like appearance [2]. The late stage of this disease shows dense fibrosis extending into the underlying muscles, fibrous bands in the buccal mucosa, lip, or palate leading to progressive restriction of maximum mouth opening (MMO), which can further cause problems with oral hygiene, speaking, and chewing [3, 4].

The etiology of OSF has not yet been fully elucidated but may be related to betel nut chewing, capsaicin, autoimmunity, allergies, genetic predisposition, and chronic vitamin and micronutrient deficiencies [5, 6]. The current mainstream view is that betel nut chewing is closely associated with the occurrence of OSF [7, 8]. Excessive use of betel nut, collagen synthesis increases and collagen degradation decreases, increased collagen cross-linking, and insufficient collagen phagocytosis and fibrokine action [9], this eventually leads to OSF. Moreover, the long-term use of betel nut can induce the production of free radicals and reactive oxygen species, leading to a high rate of oxidation/peroxidation of unsaturated fatty acids, which affects the essential components of cell membranes [10]. The study showed that the composition of betel nut products, the frequency and duration of betel nut consumption may all affect the malignant transformation rate of the disease [11] Currently, the treatment of OSF mainly includes physical therapy, hyperbaric oxygen therapy, drug therapy, and surgical treatment [12]. Drug therapy is the most common treatment, and available drug treatments include steroids, exogenous enzymes, multivitamins and micronutrients, peripheral vasodilators, human placental extracts, and other therapeutic agents. Although various treatments have been proposed for OSF over the past few decades, satisfactory results have not been achieved with most methods.

In 2012, Chole et al. [13] reviewed the literature on drug treatment for OSF to identify the role of various drugs in the treatment of OSF. In 2020, More et al. [14] summarized the clinical studies of OSF drugs in the past decade according to the mechanism of action of different drugs and targeted pathways, and discussed other potential drugs. With the emergence of new drugs for the treatment of OSF, we summarize the available pharmacological interventions for the treatment of OSF, describe the efficacy of contemporary and newly developed treatment modalities attempts to provide reference strategies for future research. Therefore, in this review, we collected relevant studies conducted in the past 10 years from 2012 to the present to systematically identify published randomized controlled trials (RCTs), clinical trials (CCTs) on various drugs for the treatment of OSF since December 2011 and update the literature with new clinical studies.

## Materials and methods

This systematic review is in accordance with the PRISMA protocols and the protocol was registered in PROSPERO with the code CRD42023429093. The PICO (Population, Intervention, Comparison, Outcome) framework was used to guide the eligibility criteria of this review. P = Patients with OSF I = Any drugs with the aim to treat OSF, C = Other drug or placebo for OSF, O = improving symptoms of OSF, such as MMO, burning sensation (BS), cheek flexibility (CF), tongue protrusion (TP).

### Search strategy

Detailed literature searches of PubMed, Web of Science, and Cochrane Library from December 2011 to September 2022 were conducted. In addition, we performed a manual search for other references in published reviews. The search strategy was based on the recommendations of the Oxford Centre for Evidence-Based Medicine and performed using subject headings, free-text terms for OSF, and relevant interventions to identify relevant RCTs, clinical trials, and meta-analyses. The detailed search strategy was (((“Oral Submucous Fibrosis“[Mesh]) OR ((submucous fibrosis) OR (submucous fibroses))) AND ((“randomized controlled trial*” OR “randomised controlled trial*” OR “randomized” OR “controlled trial”) OR (clinical trials))) AND ((“Drug Therapy“[Mesh]) OR (treatment) OR (therapy*) OR (management))

### Screening and data extraction

Articles were independently screened and extracted by two authors (X. C. and H. X.). The included studies were identified from the databases and other sources according to the previously search strategy. Other sources were references of included studies identified from databases. The screening contents included: first, a rapid title screening was performed on all the retrieved studies and irrelevant studies were excluded. Second, detailed abstracts were screened according to the defined inclusion criteria of all relevant articles. Finally, the full texts of the included studies that met the criteria were read in detail. Any disagreements between the two independent screeners were resolved by discussion and referral with another more senior and experienced member of the review team (J. G.).

### Inclusion criteria

1)Type of study: RCT, CCT were included; 2) Subjects: patients diagnosed with OSF according to pathological diagnosis or clinical manifestations, regardless of gender and race; 3) Intervention: the experimental group was treated with drugs; 4) The study was conducted from December 2011 to September 2022; 5) Studies were reported in English only.

### Exclusion criteria

(1) incomplete data (2) full text not available (3) Studies with unclear evaluation criteria; (4) Cannot be obtained Bureau index data, literature with incomplete data and wrong data.

### Quality assessment

In this review, quality was assessed independently and in duplicate by two independent reviewers using a standardized critical appraisal method for quality evaluation. The quality of RCTs was assessed using the Cochrane Risk of Bias tool, and CCTs were assessed using the tool of ROBINS. Overall, a study was judged to have a high risk of bias if at least one domain of bias was judged to be high risk. The quality of the findings generated by our review was classified as high, moderate, low, or very low in accordance with the Grading of Recommendations Assessment, Development, and Evaluation (GRADE) system [15]. It was judged by two authors (X. C. and H. X.) and checked by another author (J. G.).

### Data assessment

The data synthesis results were organized and summarized in tabular form, and the effectiveness of different drugs in the treatment of OSF were evaluated. The evaluation of BS, MMO, CF and TP were included.

## Results

**Fig. 1:**
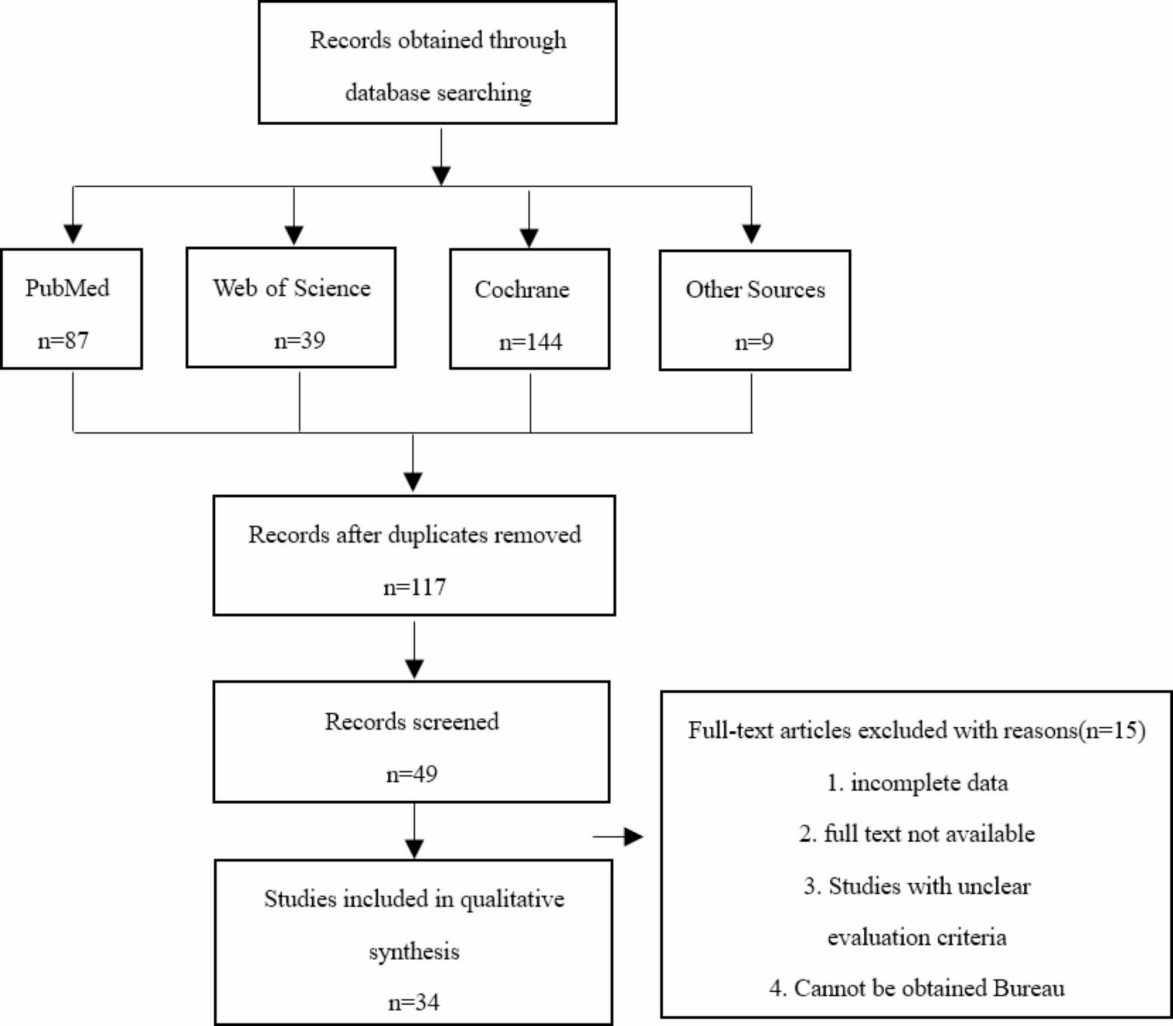
Search Strategy

Figure 1 shows the process of the literature search. The database search yielded a total of 270 studies, and the manual search yielded 9 studies. After eliminating duplicate studies, 117 studies were selected for further title and abstract screening. After screening, 68 studies were excluded as they did not meet the inclusion criteria. The remaining 49 studies were selected for full-text screening, and among the 49 studies, 34 met the criteria and were accepted. These 34 studies included 29 RCTs, 5 CCTs, a total of 2136 patients were included in the study. Four studies reported the use of steroids, it mainly includes medium-acting (triamcinolone acetonide) and long-acting (dexamethasone and betamethasone) steroid. Three reported the use of hyaluronidase. Four studies reported the use of peripheral vasodilators, including isoxsuprine and pentoxifylline. The use of antioxidants was also found to be very common: three studies reported the use of spirulina, seven studies reported the use of curcumin, and six studies reported the use of lycopene, these antioxidants were mainly used as capsules, tablets, and gels. One study used salvianolic acid B, a substance extracted from salvianolic miltiorrhiza, which is a common hemorheological agent and has the effect of promoting blood circulation and removing blood stasis. In addition, other drugs such as omega 3, allicin, colchicine, and oxitard were used to treat OSF. Oxitard capsules contains the extracts of Mangifera indica, Withania somnifera, Daucus carota, Glycyrrhiza glabra, Vitis vinifera, powders of Emblica officinalis and Yashada bhasma, and oils of Triticum sativum, these components all have anti-inflammatory effects. Table 1 presents the results of all the included studies. The included studies were all published between 2011 and 2022 and mainly reported data from Asia including China and India. The minimum duration of the intervention was 1.5 months and the maximum duration was 10 months. More than 70% of the studies presented outcomes of BS, 94% of the studies presented outcomes of MMO. Other outcomes included CF, TP, ulceration improved, blanching of oral mucosa, fibrotic bands improved, ankyloglossia improved, color of mucosa, VAS with spicy food. The results shows that drugs with evidence high quality were salvia miltiorrhiza injection (SMI) combined with triamcinolone acetonide, lycopene, pentoxifylline, curcumin, and aloe vera, and those with evidence moderate quality were allicin, colchicine, omega 3, and oxitard.

**Table 1:** Characteristics of the studies included

Study	Study design	Sample (n)	Therapeutic agents	Duration(mouths)	Outcomes (Improvement in signs/symptoms)	p value	Level of evidence
Raizada, M. K. et al. (2022)	RCT	24	biweekly intralesional injections of dexamethasone 1.5 ml and hyaluronidase 1500 IU mixed with lignocaine for 6 weeks and the placebo for 3 months	3	MMO 3.79 ± 1.07 mm TP 1.87 ± 1.54 mm CF 2.08 ± 1.38 mm BS -2.5 ± 0.78	MMO p = 0.019 TP p = 0.044 CF p = 0.035 BS p < 0.05	Moderate
		24	biweekly intralesional injections of dexamethasone 1.5 ml and hyaluronidase 1500 IU mixed with lignocaine along with 3 gm of omega 3 per day.	3	MMO 6.58 ± 1.24 mm TP 4.62 ± 1.78 mm CF 3.50 ± 1.84 mm BS -6.0 ± 1.144		
Bohra, A. et al. (2021)	RCT	21	Kali Haldi (2 mg), Aloe vera gel (2 mg), measuring scoop of 1 mg in a ratio of 2:2, over the lesion 3 times a day	3	BS − 75.9% MMO − 4.9 cm CF − 92.6% TP − 2.4%	BS p = 0.12 MMO p = 0.31 CF p = 0.004	Moderate
		21	injections of hydrocortisone acetate 25 mg/ml and hyaluronidase (1500 IU) weekly along with turbocort twice daily	3	BS − 57.2% MMO − 4.5 cm CF − 82% TP − 1.7%		
Nerkar Rajbhoj. et al. (2021)	RCT	30	5 mg of curcumin gel at respective site for 3–4 times a day		BS − 3.06 ± 0.944 MMO − 1.733 ± 1.048	BS p < 0.01 MMO p > 0.05	Moderate
		30	Aloe V era gel (aloe gel 100%)		BS − 4.500 ± 1.106 MMO − 1.367 ± 1.129		
Srivastava, R. et al. (2021)	RCT	40	curcumin lozenges three times daily	3	BS − 0.48 ± 0.506 MMO − 4.0 ± 0.981 TP − 3.85 ± 0.362	BS p <0.001 MMO p <0.001 TP p <0.001	High
		40	2 mL dexamethasone + hyaluronidase 1500 IU twice a week	3	BS − 0.38 ± 0.401 MMO − 3.28 ± 0.552 TP − 3.00 ± 0.14		
Chandrashekar, A. et al. (2021)	RCT	20	Curcumin gel. Approximately 5 mg (quantified by a scoop) of the gel was to be applied to bilateral buccal mucosa twice daily, after food intake.	2	BS − 100% MMO − 5.45 ± 1.64 mm CF − 2.65 ± 0.57 mm TP − 5.05 ± 2.13 mm	BS p < 0.01 MMO p < 0.01 CF p < 0 0.01 TP p > 0.05	Moderate
		20	Curcumin mucoadhesive patch. One patch was to be placed on the right and left buccal mucosa each,8 weeks twice daily after food intake.	2	BS − 100% MMO − 5.9 ± 2.00 mm CF − 2.66 ± 0.53 mm TP − 3.45 ± 2.52 mm		
Datarkar et al. (2020)	RCT	32	prednisolone mouthwash and antioxidant capsule	9	MMO − 10.46 mm Relief of BS - within 12.81 days Relief of recurrent ulceration - within 10.93 days	p < 0.001	High
		32	antioxidant capsule	9	MMO − 1.04 mm Relief of BS - within 21.56 days Relief of recurrent ulceration - within 20.06 days		
Lanjekar, A. B. et al. (2020)	RCT	40	1% curcumin mucoadhesive gel over the lesion on the affected mucosa 3 times daily	1.5	MMO − 51.813 BS − 60.563 Color of Oral Mucosa − 0.38 ± 0.49	MMO p < 0.001 BS p = 0.6482	High
		40	triamcinolone acetonide and hyaluronidase mucoadhesive semisolid gel 3 times daily	1.5	MMO − 44.250 BS − 63.950 Color of Oral Mucosa − 0.25 ± 0.44		
		40	professionally prepared mucoadhesive semisolid the gel of curcumin along with triamcinolone and hyaluronidase mucoadhesive semisolid gel on the affected mucosa 3 times daily	1.5	MMO − 85.438 BS − 56.988 Color of Oral Mucosa − 0.68 ± 0.47		
Rai, A. et al. (2019)	RCT	49	Antioxidant (Capsule S M Fibro; WARREN NXGEN DIVISION, Indoco Remedies Limited, Mumbai, Maharashtra, India) twice daily	3	BS − 71.32% MMO − 22.51% TP − 28.1%	BS A, p = 0.0448 B, p = 0.0262 C, p = 0.0049 MMO A, p = 0.0011 B, p = 0.003 C, p < 0.001 TP A, p < 0.001 B, p = 0.0177 C, p = 0.0314	High
		49	Turmix Tablet (tablet containing curcumin 300 mg and piperine 5 mg; Sanat Products Ltd., Bulandshahar, Uttar Pradesh, India) 3 times per day	3	BS − 73.58% MMO − 17.07% TP − 28.12%		
		49	Turmix Tablet (tablet containing curcumin 300 mg and piperine 5 mg; Sanat Products) 3 times per day along with Turmix Mouthwash (Sanat Products) 2 times per day for 12 weeks	3	BS − 90% MMO − 21.26% TP − 34.51%		
Piyush. et al. (2019)	RCT	30	a tablet with a combination of Curcumin and Piperine 300 mg 1 tablet two times daily	6	BS − 4.8 ± 2.6 MMO − 3.95 ± 4.9 CF − 0.36 ± 0.71 TP − 5 ± 7.2%	BS p = 0.008 MMO p = 0.0001 CF p = 0.245 TP p = 0.002	High
		30	Lycopene capsules 8 mg 1 tablet two times daily	6	BS − 5 ± 2.3 MMO − 4.1 ± 4.2 CF − 0.66 ± 0.80 TP − 2.4 ± 3.4		
		30	Placebo capsules	6	BS − 1.5 ± 1.5 MMO − 1.4 ± 2.3 CF − 0.03 ± 0.55 TP − 2.3 ± 4.3		
Joseph J. et al. (2019)	CCT	15	LycoRed 16 mg daily in two equally divided dose	3	MMO − 100% BS -100% partial response	p = 0.002	Moderate
		15	LycoRed along with Hyaluronidase intralesional injection 1500 IU twice weekly	3	MMO − 100% BS -92.8% partial response, 7.2% complete response		
		15	placebo capsules	3	MMO − 46.2% BS -38.5% partial response, 30.8% stable, 30.8% progression		
Tp B et al. (2019)	RCT	20	Lycopene capsule of 2 mg per day	3	MMO − 23.88 ± 0.66	p < 0.001	High
		20	Lycopene capsule of 2 mg per day for 3 months and intralesional injection of 0.5 mL of local anesthesia with 2 mL of dexamethasone twice weekly	3	MMO − 25.12 ± 0.91		
		20	intralesional injection of 0.5 mL of local anesthesia with 2 mL of dexamethasone and 1500 I.U of hyaluronidase biweekly	3	MMO − 26.43 ± 0.22		
Saran. et al.(2018)	RCT	30	4 mg of lycopene thrice daily	3	MMO − 11.1 ± 1.0%	p = 0.0001	High
		30	300 mg of curcumin thrice daily	3	MMO − 6.2 ± 0.4%		
Anuradha, A. et al. (2017)	CCT	37	drink 30 ml of aloe vera juice twice daily before food and apply 5 mg (apprx 1 scoop) of aloe vera gel over the lesion 3 times per day	3	BS − 13.3% MMO − 4.9% CF − 13.7%	BS p = 0.12 MMO p = 0.31 CF p < 0.001	High
		37	intralesional injections of hydrocortisone acetate 25 mg/ml and hyaluronidase (1500 IU) weekly	3	BS − 47.5% MMO − 5.0% CF − 12.3%		
Sadaksharam, J. et al. (2017)	RCT	15	oral pentoxifylline 400 mg thrice daily	6	MMO − 4.53 ± 1.18723 BS − 0.00 ± 0.00 submucosal thickness − 0.494 ± 0.146 mm	p < 0.00001	High
		15	2 ml of dexamethasone and 1500 I.U of hyaluronidase biweekly for 6 weeks	6	MMO − 2.73 ± 0.70373 BS − 0.13 ± 0.35 submucosal thickness − 0.598 ± 0.181 mm		
Pipalia. et al. (2016)	RCT	20	turmeric (400 mg) with black pepper(100 mg), 2 capsules TID	3	BS − 87.90% MMO − 14.37% CF − 29.03% TP − 7.98%	BS p < 0.01 MMO p < 0.01 CF p < 0.01 TP p < 0.05	High
		20	nigella sativa, 2 capsules of 500 mg TID	3	BS − 78.91% MMO − 13.75% CF − 44.12% TP − 8.95%		
Singh. et al. (2016)	RCT	19	Aloe vera gel on each side of the oral mucosa three times daily	3	BS − 93.8% MMO − 9.1% TP − 3.9%	BS p = 0.001 MMO p = 0.004 TP p = 0.001	High
		18	antioxidant capsules twice daily	3	BS − 71.2% MMO − 5.3% TP − 2.2%		
Patil, S. et al. (2015)	RCT	60	2 oxitard capsules twice daily	3	MMO Baseline 19.1 ± 2.4 After 3 months 31.5 ± 2.9 TP Baseline 10.1 ± 1.4 After 3 months 24.5 ± 2.5 BS Baseline 60 After 3 months 3	MMO p < 0.001 TP p < 0.001 BS p = 0.0001	High
		60	placebo tablets twice daily	3	MMO Baseline 20.1 ± 2.1 After 3 months 23.1 ± 1.9 TP Baseline 9.3 ± 2.2 After 3 months 22.1 ± 1.8 BS Baseline 60 After 3 months 24		
Jiang. et al. (2015)	RCT	24	intralesional injection of triamcinolone acetonide (2 mg)	4	MMO − 2.27 ± 0.84 mm BS − 2.79 ± 0.87	MMO P < 0.001 BS P < 0.001	High
		24	intralesional injection of allicin (1 mg)	4	MMO − 5.16 ± 1.04 mm BS − 4.33 ± 1.04		
Hazarey, V. K. et al. (2015)	RCT	15	Longvida lozenges (Mfg Lic.: GA/1482) (400 mg lozenges) 2 g/day	3	MMO − 5.93 ± 2.37 mm VAS with normal food − 36 (24-65.5) VAS with spicy food − 45 (37.5–74.5)	MMO p < 0.0001 VAS with normal food p = 0.0007 VAS with spicy food p = 0.0007	High
		15	topical steroid 3 times daily	3	MMO − 2.66 ± 1.76 mm VAS with normal food − 15 (7-26.5) VAS with spicy food − 23 (13.5–31.5)		
Patil, S.et al. (2015)	RCT	21	500 mg spirulina in 2 divided doses	3	MMO Baseline 19.9 ± 2.1 After 3 months 25.8 ± 2.5	p < 0.05	High
		21	5 mg aloe vera gel to be applied topically thrice daily	3	MMO Baseline 19.1 ± 2.7 After 3 months 23.9 ± 1.9		
Prabhu, N. et al. (2015)	RCT	15	A:Pentoxifylline 2 tablets daily	4	On the assessment of MMO and TP, there was no significant improvement in either of the groups individually or in comparison. But both groups showed quite a significant improvement individually in BS	MMO A, p = 0.2077; B, p = 0.1437 TP A, p = 0.8123; B, p = 0.1352 BS A, p = 0.0423; B, p = 0.0117	High
		15	B:placebo tablets	4			
James, L. et al. (2015)	CCT	28	hyaluronidase 1500 IU mixed in 1.5 ml of dexamethasone and 0.5 ml of lignocaine HCL injected intralesionally biweekly for 4 weeks	9	Limited MMO − 92.85% BS − 89.28% Painful ulceration − 78.57% Blanching of oral mucosa − 71.42%	/	Moderate
Alora R et al. (2015)	RCT	15	hyaluronidase (1500 I.U)	6	BS − 2.60 ± 1.60 Pain while opening mouth − 1.00 ± 1.69 Tightness of mucosa − 3.73 ± 1.67 MMO − 6.67 ± 3.74	BS p = 0.520 Pain while opening mouth p = 0.035 Tightness of mucosa p = 0.008 MMO p = 0.068	High
		15	dexamethasone (8 mg)	6	BS − 3.20 ± 1.52 Pain while opening mouth − 0.33 ± 0.72 Tightness of mucosa − 2.00 ± 1.51 MMO − 4.27 ± 1.58		
		15	hyaluronidase (750 I.U) + dexamethasone 4 mg	6	BS − 2.80 ± 1.20 Pain while opening mouth − 1.93 ± 2.15 Tightness of mucosa − 2.53 ± 1.19 MMO − 5.80 ± 2.60		
Goel, S et al. (2015)	CCT	90	Control group	6	Stage I MMO 0.00 ± 0.00 (range 1–5 mm) stage II MMO 0.00 ± 0.00 (range 3–9 mm) stage III MMO 0.00 ± 0.00 (range 4–10 mm)	Stage I p > 0.05 Stage II p < 0.0001 Stage III p < 0.0001	Moderate
		90	Lycopene group (4 mg/day)	6	Stage I MMO 3.00 ± 1.11 (range 1–5 mm) stage II MMO 6.07 ± 2.00 (range 3–9 mm) stage III MMO 6.53 ± 1.45 (range 4–10 mm)		
		90	Betamethasone group (4 mg/ml weekly	6	Stage I MMO 3.30 ± 1.51 (range 1–5 mm) stage II MMO 9.47 ± 2.47 (range 5–14 mm) stage III MMO 3.27 ± 1.36 (range 1–5 mm)		
Yadav, M. et al. (2014)	RCT	20	4 mg Dexamethasone & 1500 I.U Hyaluronidase	3	BS − 15.6 (11.2) Interincisal distance − 1.5 (1) TP − 0.9 (0.9)	BS p < 0.0001 Interincisal distance p = 0.0877 TP p = 0.0195	High
		20	Two Curcumin tablets (Turmix 300 mg) per day	3	BS − 0 (0) Interincisal distance − 0.82 (1.1) TP − 0.23 (0.66)		
Krishnamoorthy, B. et al. (2013)	RCT	25	colchicine 0.5 mg twice daily + injection of Hyaluronidase 1,500 IU was mixed in 1 ml of lignocaine. 0.5 ml	3	33% in group 1 got relief in the second the week itself as against 21% in group 2	P < 0.05	High
		25	intralesional injection of Hyaluronidase 1,500 IU and 0.5 ml of injection Hydrocortisone acetate 25 mg/ml in each buccal mucosa once a week	3			
Alam, S. et al. (2013)	RCT	30	medicinal treatment + aloe vera gel over the buccal mucosa, palate, the retromolar region, and the floor of the mouth twice daily	3	BS 5 ± 0 to 1.73 ± 1.01 MMO − 13.74 mm TP 26.00 ± 5.83 mm to 31.67 ± 6.66 mm	BS p < 0.01 MMO p < 0.01 TP p > 0.05	High
			surgical treatment + aloe vera gel over the buccal mucosa, palate, the retromolar region, and the floor of the mouth twice daily	3	BS 5 ± 0 to 3.66 ± 0.97 MMO 37.46 ± 2.50 mm to 38.93 ± 3.32 mm TP 22.86 ± 5.08 mm to 27.10 ± 2.46 cm		
		30	medicinal treatment + No aloe vera gel	3	BS 5 ± 0 to 3.53 ± 1.17 MMO 24.0 ± 7.53 to 30.0 ± 7.41 mm TP 32.46 ± 6.35 mm to 36.66 ± 5.31 mm		
			surgical treatment + No aloe vera gel	3	BS 5 ± 0 to 4.23 ± 0.75 MMO 37.33 ± 2.12 to 34.0 ± 3.18 TP 24.2 ± 5.83 mm to 27.53 ± 2.81 cm		
Mulk, B. S., et al. (2013)	RCT	20	pentoxifylline 400 mg twice daily	4	MMO − 0.30 ± 0.0725 BS − 4.45 ± 1.191 TP − 0.18 ± 0.089	MMO p = 0.35 BS p = 0.04 TP p = 0.25	High
		20	spirulina capsules 0.5gm twice daily	4	MMO − 0.36 ± 0.27 BS − 5.40 ± 1.353 TP − 0.16 ± 0.1095		
Shetty, P. et al. (2013)	CCT	20	antioxidants (Spirulina 500 mg) orally twice daily for 3 months + biweekly treated with steroid injection (Betamethasone 4 mg/ml)	3	MMO − 5.7500 ± 2.73140 BS − 4.7000 ± 1.65752	MMO p = 0.001 BS p < 0.001	Moderate
		20	placebo capsules daily two times for 3 months + biweekly treated with steroid injection (Betamethasone 4 mg/ml)	3	MMO − 2.8500 ± 1.34849 BS − 2.6500 ± 1.38697		
Bhadage, C. J. et al. (2013)	RCT	15	A:10 mg isoxsuprine tablets four times per day	1.5	BS − 0.67 ± 1.80 MMO − 29.5 ± 8.9	BS A, p < 0.00001; B, p < 0.00001; C, p = 0.003; MMO A, p < 0.00001; B, p < 0.00001; C, p = 0.006;	High
		15	B:Biweekly 2ml dexamethasone with 1500 IU hyaluronidase intralesional injections	1.5	BS − 0.00 ± 0.00 MMO − 26.9 ± 3.1		
		10	C:placebo tablets	1.5	BS − 4.60 ± 1.20 MMO − 26.9 ± 3.1		
Jiang, X. W et al. (2013)	RCT	14	triamcinolone acetonide (2 mg)	10	MMO − 2.00 ± 1.21 mm BS − 3.05 ± 0.76	p < 0.05	High
		14	salvianolic acid B (4 mg)	10	MMO − 3.48 ± 2.23 mm BS − 4.96 ± 0.97		
		14	TA (2 mg) and SA-B (4 mg)	10	MMO − 5.50 ± 1.80 mm BS − 6.11 ± 0.93		
Karemore. et al. (2012)	RCT	46	8 mg softgel Lycored TM orally per day in two divided doses of 4 mg	3	There was a significant difference in maximum MMO between the study and placebo group with Z calculated value of 5.56 mm at the exit in maximum MMO. Wherein the lycopene group showed a significant decrease in post-treatment juxta-epithelial collagen deposition and chronic inflammatory infiltrate	p < 0.05	High
Sudarshan. et al. (2012)	RCT	10	5 mg of Aloe vera gel on each side of the buccal mucosa three times daily	3	BS − 58.0 ± 18.7 MMO − 5.1 ± 2.5 CF − 0.06 ± 0.05 TP − 3.1 ± 2.2	BS p = 0.008 MMO p = 0.02 CF p = 0.011 TP p = 0.08	High
		10	antioxidant capsules twice daily	3	BS − 36.5 ± 12.9 MMO − 2.5 ± 1.9 CF − 0.00 ± 0.06 TP -1 0.7 ± 1.0		
Mehrotra, R. et al. (2011)	RCT	30	placebo	7	BS − 39.4% Ulceration improved − 35.5% Ankyloglossia improved − 22.6% Fibrotic bands improved − 19.5% Improvement in trismus − 6 mm	p < 0.05	High
		32	400 mg. Pentoxifylline for a period of 7 months	7	BS − 86.6% Ulceration improved − 84.1% Ankyloglossia improved − 39.3% Fibrotic bands improved − 32.9% Improvement in trismus − 10 mm		

MMO: maximum mouth opening, BS: burning sensation, CF: cheek flexibility, TP: tongue protrusion

### Risk of bias in RCT

**Fig. 2:**
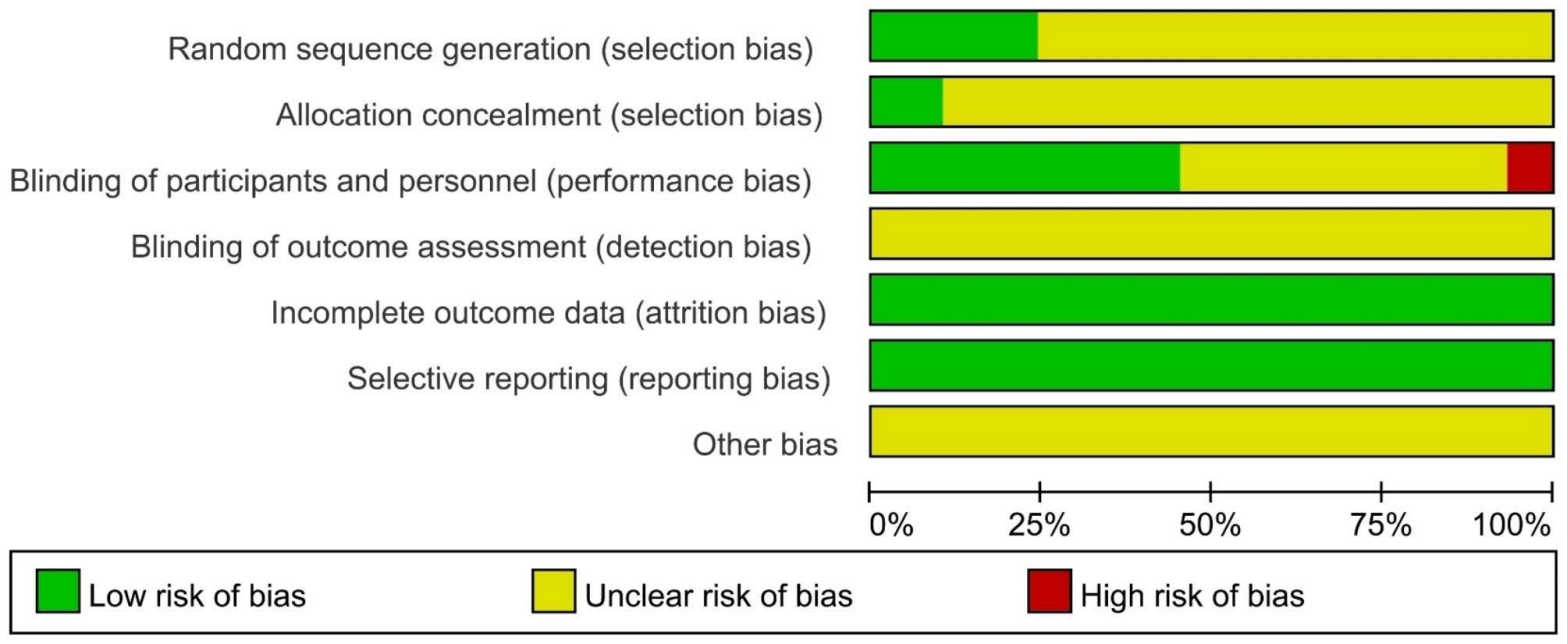
Risk of bias in randomized controlled trials

An analysis of the risk of bias in RCTs found that two studies [16, 17] were rated as having a high risk of bias due to the open-label approach used during the trial, which influenced both objective and subjective outcomes. The remaining studies included all had moderate risk scores for bias. The parameters that led to bias were: no mention of randomization in detail, lack of assignment hiding and blind procedures, and use of open labels for trials. Only 7 studies [18–24] mentioned the details of random sequence production, 3 studies [19, 22, 25] used allocation concealment, and only 12 studies [18, 19, 21, 22, 24, 26–32] used blind methods. The results were shown in Fig. 2.

### Risk of bias in CCT

**Fig. 3:**
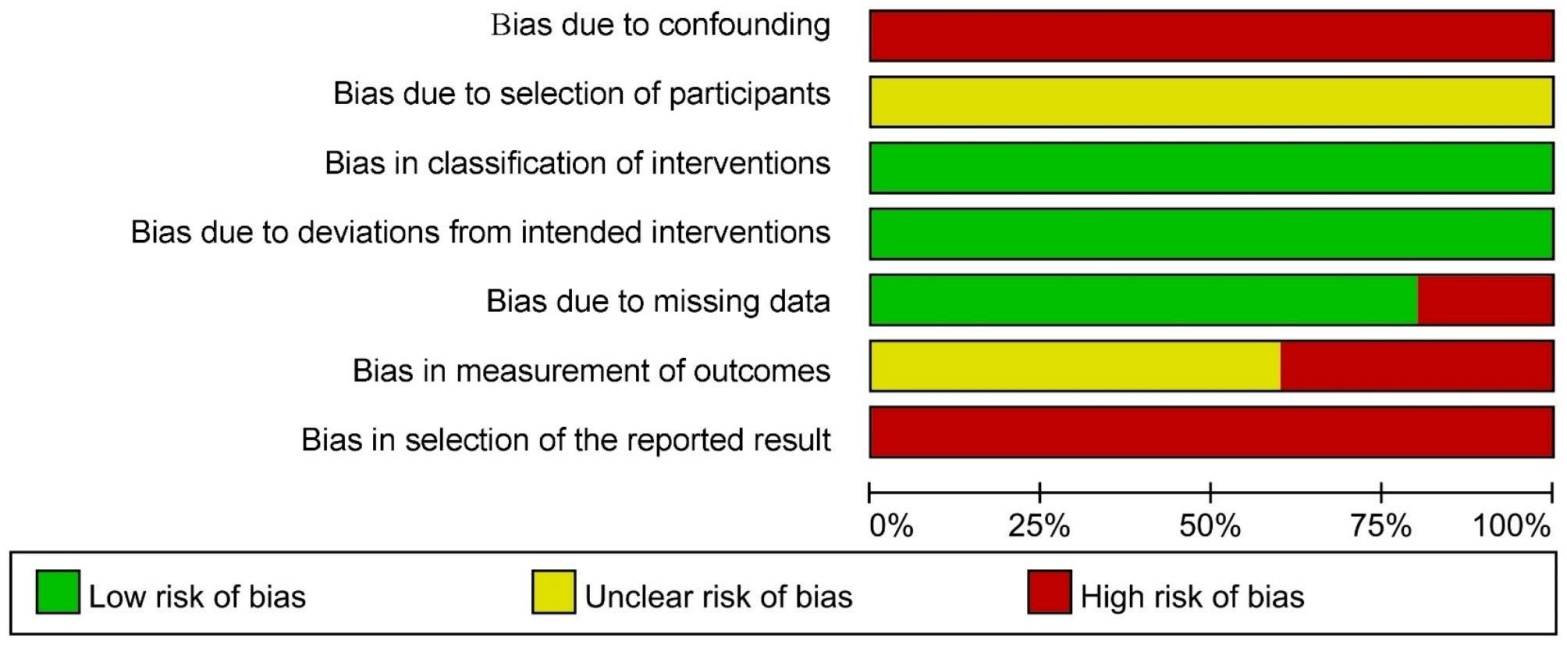
Risk of bias in clinical trials

The risk of bias score in 5 CCTs [33–37] was high, which was due to the lack of control for confounding factors, and the bias in measurement and outcome reporting. Considering that the 5 studies did not achieve serious bias and had some reference value, these 5 clinical trials were included in the review for analysis. The results were shown in Fig. 3.

### Side-effects

No significant serious side effects were found among the included drugs. In one study, the use of oxitard capsules caused mild abdominal discomfort, and in another study, patients reported mild pain from intralesial injections of dexamethasone [38]. In one study, it was suggested that high-dose isoxsuprine may cause side effects such as facial flushing, high-dose tachycardia, and hypotension, the study used normal dosing, so no related side effects occurred [39]. Mild adverse effects can also occur when curcumin is not used properly, it can cause indigestion, abdominal pain, abdominal distension, dizziness, and other minor adverse reactions. Therefore, more attention should be paid to its adverse reactions to ensure that curcumin is effective and safe for the treatment of OSF.

## Discussion

**Fig. 4:**
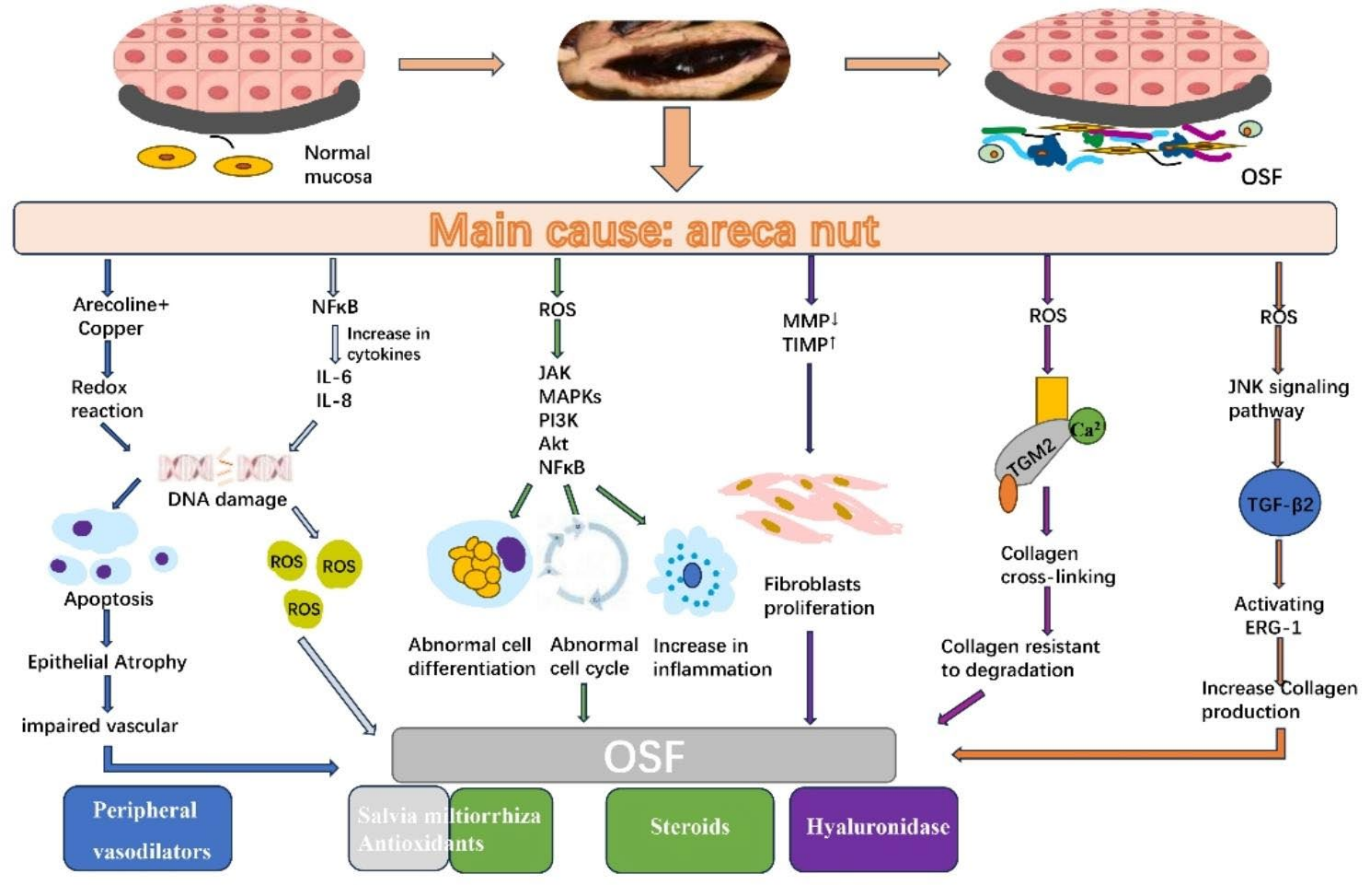
Pathogenesis of OSF and targeted drug therapies

OSF is a chronic disease, and betel nut is the main pathogenic factor. In addition, smoking, nutritional deficiencies, genetic and immune processes are other potential factors. The pathogenesis of OSF and the various interventions that work against different pathogenesis are shown in Fig. 4. The treatment of OSF is difficult, and the current treatment methods are mainly aimed at relieving the signs and symptoms of the disease. Usually, OSF is treated with drugs in the early stages, followed by physical therapy in severe cases and surgery in the late or advanced stages. Many studies have shown that a combination of drugs, including steroids, enzymes, antioxidants, multivitamins, and minerals, can alleviate the signs and symptoms of OSF; however, these studies lacked solid evidence or the sample size was too small to be representative, and related drug therapy trials have been disappointing. Therefore, it is fairly challenging to compare or even combine their effects in a scientifically meaningful manner. Therefore, our article reviewed RCTs and clinical trials for a better assessment of the available evidence.

### Steroids

Owing to their immunosuppressive and anti-inflammatory properties, steroids are widely used to treat OSF. In terms of immunosuppression, steroids can activate sensitized lymphocytes against specific antigens to release soluble factors. In terms of their anti-inflammatory effects, steroids can inhibit the proliferation of inflammatory factors and increase the apoptosis of inflammatory cells. Several glucocorticoids, such as short-acting (hydrocortisone), medium-acting (triamcinolone), and long-acting (dexamethasone and betamethasone), have been used for the treatment of OSF [40]. In the early stage, steroids can effectively improve MMO and BS; however, they do not improve the abnormal accumulation of fibrotic tissue [33]. Therefore, steroids alone cannot completely improve the quality of life of patients with OSF. Two studies from Yadav et al. [23] and Anuradha et al. [36] showed that dexamethasone combined with hyaluronic acid was superior to curcumin and aloe vera in improving BS and MMO, respectively. Datarkar et al. [18] showed that prednisolone mouthwash was superior to antioxidant capsules only in improving MMO, but was inferior to antioxidant capsules in relieving BS and ulcers. Systemic corticosteroids are rarely used in the treatment of OSF and usually administered as intrafocal injections or mouthwashes [41]. Overall, steroids can alleviate the signs and symptoms of OSF and can be used in combination with other medications as adjunctive therapy.

### Salvia miltiorrhiza

SMI has an excellent antifibrotic activity in vitro. Salvianolic acid B (SA-B) is the important active compound of SMI, it can inhibit collagen accumulation and procollagen gene transcription by targeting the mitogen-activated protein kinase/extracellular signal-regulated kinase pathway, Akt pathway and transforming growth factor /Smad pathway [42]. Jiang et al. [43] compared the efficacy of SA-B combined with triamcinolone acetonide in the treatment of OSF. The SA-B group achieved better improvement in the MMO and BS. A meta-analysis by Guo et al. [44] concluded that in combination with steroids, SMI can effectively improve the subjective symptoms of MMO and BS in patients with OSF and reduce the area of oral mucosal lesions without causing adverse reactions.

### Enzymes

Hyaluronidase was originally found in bacteria and widely distributed in nature. It can break down hyaluronic acid (an important component of the extracellular matrix), which reduces the viscosity of intracellular cement and collagen formation. A significant feature of OSF is the massive abnormal deposition of collagen fibers and reduced fibrinolysis [45]. Exogenous enzyme hyaluronidase can target MMP-1 or MMP-2 to destroy abnormal fibrotic tissue, thereby alleviating or curing OSF [46, 47]. Study of Beenakumary et al. showed that hyaluronidase combination with dexamethasone injection was superior to lycopene in improving MMO [41]. Johny et al. [34] showed that LycoRed along with hyaluronidase intralesional injection had 92.8% partial response and 7.2% complete response in improving BS. However, LycoRed alone only achieved a partial response. Hyaluronidase could effectively improve MMO and reduce mucosal tightness, compensating for the lack of effect of steroids in improving the abnormal accumulation of fibrotic tissue; therefore, the two drugs are often used in combination [27]. A meta-analysis by Guo et al. [48] showed that hyaluronidase combined with steroids showed a significantly better effect than placebo in alleviating the BS and improving the MMO in OSF and was as effective as control drugs (such as aloe vera, pentoxifylline, and lycopene).

### Peripheral vasodilators

A potential cause of OSF is the progressive loss of diseased mucosal blood vessels, which can lead to epithelial atrophy [49]. Therefore, vasodilators are considered effective for the treatment of OSF. Pentoxifylline (PTX), a trisubstituted methylxanthine derivative, is used as a vasodilator and is effective in the treatment of diseases caused by chronic peripheral arterial occlusion [50]. As mucosal ischemia and the resulting epithelial atrophy may be factors in the pathogenesis of OSF, PTX can be therapeutic owing to its ability to relax and dilate blood vessels, ensuring increased blood supply to the ischemic tissue and enabling nutritional and therapeutic drugs to reach the affected tissue [51]. Isoxsuprine is a phenylalanine derivative of epinephrine and a β-adrenoreceptor agonist that causes an overall increase in the cytosolic calcium concentration, stimulating the production and release of several endothelium-derived vasodilators [52]. In a clinical trial, isoxsuprine plus physical therapy was superior to placebo plus physical therapy in reducing BS, but there was no difference in improving MMO [39]. Sadakshara et al. [28] showed that oral pentoxifylline 400 mg thrice daily was superior to dexamethasone combine with hyaluronidase in improving MMO. Prabhu et al. [53] and Mehrotra et al [29] suggest that oral pentoxifylline was effective in improving BS and fibrotic band. A meta-analysis by Liu et al. [54] showed that PTX is an effective treatment as it can not only increase MMO of patients with OSF but also relieve BS in the mouth. High-dose isoxsuprine may cause facial flushing, high-dose tachycardia, hypotension and other side effects, which can be effectively prevented under normal use doses. Thus, 400 mg twice daily PTX is well tolerated and is expected to become more effective over time.

### Antioxidants

One of the pathogenic mechanisms of betel nut is the production of reactive oxygen species, free radicals, and peroxidase to destroy the cellular structure. Based on this hypothesis, several studies have tested various natural or synthetic antioxidants and reported that using them can improve the condition of betel nut damage to the mouth. The mechanism of action of antioxidants is to inhibit ROS production by targeting JAK, MAPK, PI3K and other pathways, and can also inhibit inflammatory response by targeting pro-inflammatory mediators, such as NF-κB, ROS, COX-2, IL-1, IL-2, TGF-β, growth factors, apoptotic proteins, receptors and various kinases [55, 56]. Natural antioxidants include lycopene, aloe vera, curcumin, and spirulina.

### Lycopene

Lycopene is a carotenoid found in vegetables and red fruits such as tomatoes, watermelon, and papaya [57]. Lycopene is a carotenoid with strong antioxidant properties because of its high singlet oxygen quenching ability and the ability to quench other free radicals in vitro; it also has antioxidant properties [57]. Many studies have shown that lycopene can prevent and treat diseases, such as oral diseases, heart failure and tumors, through anti-inflammatory, antioxidant and anti-proliferative activities [58–60]. Karemore et al [26]. showed that compared with the placebo group, the lycopene group had significantly improved MMO, significantly reduced para-epithelial collagen deposition and chronic inflammatory infiltration after treatment. Saran et al [25]. showed that the lycopene group was superior to the curcumin group in improving MMO. In a study by Piyush et al. [19], lycopene showed superior therapeutic effects in terms of MMO, BS, CF, and TP in 90 patients. In addition, a meta-analysis by Guo et al. [61] showed that lycopene was more effective than placebo in improving the MMO of patients with OSF. Compared with control patients who received drugs such as aloe vera gel, curcumin, spirulina, and betamethasone, patients with OSF showed significantly improved MMO after 1, 2, and 3 months of lycopene treatment, with no significant effect on BS, tongue process, or lesion-related pain. Therefore, lycopene is a promising antioxidant for the treatment of patients with OSF, especially for improving MMO.

### Spirulina

Spirulina is a blue-green alga with abundant vitamins (A and B12), minerals, carotenoids, and phycocyanin, it is considered as superfood by the WHO. It imparts the antioxidant effect by increasing IL-2 concentration and decreasing IL-6 concentration [62]. Due to its antioxidant, anti-inflammatory, and immunomodulating properties, spirulina has shown promising results in the management of OSF. Shetty et al. [35] reported that spirulina can effectively improve MMO and BS in patients with OSF. Studies by Mulk et al. [63] showed that spirulina was more effective in improving MMO than pentoxifylline. In a RCT from Patil et al. [64] show that spirulina was superior to aloe vera gel in improving MMO.

### Aloe vera

Aloe vera is an ancient plant with a variety of pharmacological effects and has long been used as a medicine to treat many diseases without any reported side effects [65, 66]. Aloe vera is a rich source of vitamins, enzymes, minerals, and sugars, vitamins include antioxidant vitamins A, C, and E, which help neutralize free radicals and act as antioxidants. [62]. Enzymes in aloe vera help reduce inflammation, and polysaccharides promote wound healing and exhibit anti-inflammatory, anticancer, immunomodulatory, and gastric protective properties that explain their role in treating OSF. Nerkar et al. [17] reported that compared with curcumin gel, aloe vera was more effective in improving BS and MMO. Alam et al. [30] showed that the combination of aloe vera after medical or surgical treatment was more effective in improving both BS and TP than either medical or surgical treatment alone. Bohr et al. [31] showed that daily use of aloe vera gel was superior to weekly injections of hydrocortisone acetate and hyaluronidase in improving BS, MMO, TP, and CF. Two other trials from Singh et al. and Sudarshan et al. [32, 67] showed that aloe vera was superior to antioxidant capsules in improving MMO, TP, BS, and CF. A meta-analysis conducted by Al-Maweri et al. [68] showed that compared with other drugs, aloe vera was well tolerated, had few side effects, and was significantly better in reducing BS in the short-term treatment; however, differences in its long-term treatment effects and improvements in MMO, TP, and CF were not significant. Thus, short-term treatment with aloe vera can effectively improve the oral BS in patients with OSF. Therefore, topical application of aloe vera can be a convenient, economical and effective treatment for OSF without any side effects, it is a very safe therapeutic drug.

### Curcumin

Curcumin is a polyphenolic compound extracted from the rhizome of curcuma longa and tuber tubers of Zingiberaceae, it is widely used as an antioxidant in many diseases. It interacts with thioredoxin reductase to induce reactive oxygen species (ROS) and inhibit the action of nicotinamide adenine dinucleotide phosphate oxidase, which is responsible for the generation of ROS, thus playing an antioxidant role [69]. In this review, in the study of Chandrashekar et al. [20] Curcumin gel and Curcumin mucoadhesive patch were more effective in improving BS, MMO, TP and CF. In the study by Pipalia et al. [21] curcumin plus black pepper was more effective in improving BS and MMO than nigella sativa. In the study by Rai et al. [22] curcumin tablet (containing curcumin 300 mg and piperine 5 mg) was more effective than ordinary antioxidants in improving BS, MMO and TP. In addition, Lanjekar et al. [70] evaluated the efficacy of curcumin in patients with OSF and found that curcumin showed better improvement in MMO and color of oral mucosa compared with triamcinolone acetonide and hyaluronidase mucoadhesive semisolid gel, better results were obtained when the two drugs were used in combination. Therefore, a combination of curcumin, triamcinolone acetonide, and hyaluronidase is recommended as hyaluronidase facilitates deeper administration of curcumin and triamcinolone acetonide, which have synergistic effects between each other. A meta-analysis by Guo et al. [71] showed that compared with placebo, oral curcumin significantly improved the symptoms of MMO and BS. Compared with lycopene and other drugs, curcumin was less effective in improving MMO after 1 month of treatment, and the improvement of BS after 3 months of treatment was better than that in the control group. Therefore, curcumin can be used as an effective treatment for OSF.

### Other drugs

In addition to the abovementioned drugs, allicin, colchicine, omega 3, and oxitard have a certain effect on the treatment of OSF. There is evidence showing that allicin has important anti-inflammatory effects. Studies have shown that TNF-α is associated with the severity of OSF. First, allicin can reduce TNF-α protein and mRNA levels [72]. Second, allicin has antioxidant activity, which can reduce lipid peroxidation and scour hydroxyl radicals [73]. Finally, allicin can increase the expression of vascular endothelial growth factor and angiopoietin and decrease the expression of angiostatin, thereby promoting angiogenesis and accelerating self-repair [74]. Therefore, we attributed the possible therapeutic mechanism of allicin to its anti-inflammatory and antioxidant effects and the angiogenic ability of its breakdown products. One study showed that intralesional injection of allicin (1 mg) for 16 weeks can significantly improve MMO and BS compared with the control group [24]. Colchicine reduces collagen synthesis by disrupting microtubule formation and preventing the extrusion of collagen from fibroblasts [75]. Krishnamoorthy et al. [76] studied the effects of oral administration of 500 mg colchicine and intralesional injection of 0.5 ml hyaluronidase 1,500 IU in the management of OSF and found superior results with colchicine in ameliorating the symptoms of OSF. Omega 3 fatty acids are polyunsaturated essential fatty acids that humans cannot synthesize and must rely on dietary sources. They competitively inhibit the production of arachidonic acid metabolites through the cyclooxygenase and lipoxygenase pathways, thereby limiting tissue damage [77]. Studies have confirmed the inhibitory effect of omega 3 on the secretion of pro-fibrotic TGFβ1 and MMP-9 [78]. It can improve endogenous fibrinolysis and microcirculation by improving vasomotor function. Thus, it enhances vasodilatation and ameliorates the mucosal vessels that are significantly damaged by fibrosis in OSF. In their study, Raizada et al. [79] reported that omega 3 can be used as an adjunctive treatment option in patients with OSF to reduce subjective symptoms, and when combined with dexamethasone and hyaluronidase, omega 3 improved MMO, BS, and CF more effectively. Oxitard capsules are formulated using extracts of mango, morelle, carrot, licorice, grape, triglyceride powder, and wheat oil. These components have the effects of regulating immunity, antiinflammation, convergence and inhibition of BS, antioxidation, and wound healing, as well as certain curative effects on OSF for various reasons [38]. Santosh et al. [38] showed that oxitard capsules significantly improve MMO, TP, BS, pain associated with the lesion, and difficulty in swallowing and speech, with very few side effects.

In a network meta-analysis comparing the efficacy of different treatment interventions for OSF [80], most interventions were found to be superior to placebo in improving clinical symptoms, such as MMO and BS. Oxitard is superior to other interventions in improving MMO, aloe vera is superior in relieving BS, and lycopene has the lowest propensity for side effects and can be considered the best safety agent.

GRADE system was used to evaluate the evidence quality, and it was found that drugs with evidence high quality were SMI combined with triamcinolone acetonide, lycopene, pentoxifylline, curcumin, it is a good choice for the treatment of OSF.

This review had a few limitations, the first of which was the small sample size; only one clinical trial involved more than 150 people. Second, patients could only be verbally advised not to chew betel nuts during the trial. However, there was no effective means to monitor and confirm that all patients have eliminated the harmful habits and thus the results might be biased to some extent.

## Conclusion

In conclusion, our findings found that steroids, hyaluronidase, pentoxifylline, antioxidants, omega 3, colicine, and allicin can alleviate the symptoms of OSF, for long-term treatment, lycopene is effective and has few side effects. Aloe vera is the most effective for relieving the symptoms of severe burning. Regardless of the number of treatment options available, abstaining from betel nut chewing is the best strategy to prevent OSF. Our review is intended only as a reference for clinical medication management, we hope that more high-quality meta-analyses, systematic reviews and multicenter RCTs with larger samples will provide more reference suggestions for the treatment of OSF in the future.

### Electronic supplementary material

Below is the link to the electronic supplementary material.

Supplementary Material 1

## Data Availability

The datasets used and/or analysed during the current study are available from the corresponding author on reasonable request.
