# Supplementary material for: Drug treatment for oral submucous fibrosis: an update
Source: BMC Oral Health. 2023 Oct 12;23:748. doi: 10.1186/s12903-023-03488-9 (PMC10568776; doi:10.1186/s12903-023-03488-9)
Supplement: Supplementary file 1 — Supplementary Material 1 [file 12903_2023_3488_MOESM1_ESM.doc]

| **Section/topic** | **#** | **Checklist item** | **Reported on page #** |
| --- | --- | --- | --- |
| **TITLE** | | |  |
| Title | 1 | Drug treatment for oral submucous fibrosis: a systematic review | 1 |
| **ABSTRACT** | | |  |
| Structured summary | 2 | **Objective:** The aim of this review is to evaluate the different medicinal interventions available for the management of oral submucous fibrosis (OSF).  **Materials and methods:** We searched a comprehensive electronic search on PubMed, Web of Science, and Cochrane Library databases for articles related to OSF patients treated with medications from December 2011 to September 2022. GRADE system was used to evaluate the evidence quality.  **Results:** Twenty-nine randomized controlled trials (RCTs), six non-RCT studies were included, and the use of drugs for OSF treatment were evaluated. The main outcomes were improvement in mouth opening, burning sensation, cheek flexibility, and tongue protrusion. Drugs like steroids, hyaluronidase, pentoxifylline, lycopene, curcumin, dpirulina, aloe vera, omega3, oxitard, allicin, colchicine have been used. It was found that drugs with evidence high quality were SMI combined with triamcinolone acetonide, lycopene, pentoxifylline, curcumin, and aloe vera, and those with evidence moderate quality were allicin, colchicine, omega 3, and oxitard.  **Conclusion:** Based on the results of our comprehensive analysis, for long-term treatment, we found lycopene with low side effects, whereas for relieving the symptoms of severe burning sensation, aloe vera is the most effective. Although the resent review has made some progress, drug therapy for OSF remains unclear, and more high-quality RCTs are needed to identify better treatments for OSF. | 1-2 |
| **INTRODUCTION** | | |  |
| Rationale | 3 | In this review, quality was assessed independently and in duplicate by two independent reviewers using a standardized critical appraisal method for quality evaluation. All disagreements were settled through discussion, which included the participation of a third review author.  The quality of the findings generated by our review were classified as high, moderate, low, or very low in accordance with the Grading of Recommendations Assessment, Development, and Evaluation (GRADE) system(Guyatt et al., 2011). Authors independently screened the titles and abstracts of studies that fulfilled the selection criteria and checked for agreement. | 4-5 |
| Objectives | 4 | The aim of this review is to evaluate the different medicinal interventions available for the management of oral submucous fibrosis (OSF). | 1 |
| **METHODS** | | |  |
| Protocol and registration | 5 | The review protocol does not exist. | 5 |
| Eligibility criteria | 6 | **Inclusion Criteria**  1)Type of study: RCT, non-RCT studies were included; 2) Subjects: patients diagnosed with OSF according to pathological diagnosis or clinical manifestations, regardless of gender and race; 3) Intervention: the experimental group was treated with drugs; 4)The study was conducted from December 2011 to September 2022; 5) Studies were reported in English only.  **Exclusion criteria**  (1) incomplete data (2) full text not available (3) Studies with unclear evaluation criteria; (4) Cannot be obtained Bureau index data, literature with incomplete data and wrong data. | 4 |
| Information sources | 7 | Detailed literature searches of PubMed, Web of Science, and Cochrane Library from December 2011 to September 2022 were conducted. In addition, we performed a manual search for other references in published reviews. | 3 |
| Search | 8 | The search strategy was based on the recommendations of the Oxford Centre for Evidence-Based Medicine and performed using subject headings, free-text terms for OSF, and relevant interventions to identify relevant RCTs, clinical trials, and meta-analyses. The detailed search strategy was ((("Oral Submucous Fibrosis"[Mesh]) OR ((submucous fibrosis) OR (submucous fibroses))) AND (("randomized controlled trial*" OR "randomised controlled trial*" OR "randomized" OR "controlled trial") OR (clinical trials))) AND (("Drug Therapy"[Mesh]) OR (treatment) OR (therapy*) OR (management)) | 4 |
| Study selection | 9 | In the rough screening phase, articles were excluded by screening based on title and abstract. In the careful screening stage, the full text was read for screening, and after screening the full text, the selected articles were included using a predetermined data extraction form. In each step, two reviewers Jincai Guo and Xueru Chen independently screened the literature, extracted the data, and checked each other. If there were different opinions, the third reviewer Hui Xie would assist in the judgment | - |
| Data collection process | 10 | The included studies were all published between 2011 and 2022 and mainly reported data from Asia including China and India. The minimum duration of the intervention was 1.5 months and the maximum duration was 10 months. The main outcomes were improvement in mouth opening, burning sensation, cheek flexibility, and tongue protrusion. | 5 |
| Data items | 11 | mouth opening, burning sensation, cheek flexibility, and tongue protrusion. | 5 |
| Risk of bias in individual studies | 12 | Not applicable | - |
| Summary measures | 13 | Not applicable | - |
| Synthesis of results | 14 | The quality of the findings generated by our review were classified as high, moderate, low, or very low in accordance with the Grading of Recommendations Assessment, Development, and Evaluation (GRADE) system(Guyatt et al., 2011). Authors independently screened the titles and abstracts of studies that fulfilled the selection criteria and checked for agreement. | 5 |

Page 1 of 2

| **Section/topic** | **#** | **Checklist item** | **Reported on page #** |
| --- | --- | --- | --- |
| Risk of bias across studies | 15 | Not applicable | - |
| Additional analyses | 16 | Not applicable | - |
| **RESULTS** | | |  |
| Study selection | 17 | The database search yielded a total of 270 studies, and the manual search yielded 9 studies. After eliminating duplicate studies, 117 studies were selected for further title and abstract screening. After screening, 68 studies were excluded as they did not meet the inclusion criteria. The remaining 49 studies were selected for full-text screening, and among the 49 studies, 35 met the criteria and were accepted. These 35 studies included 29 RCTs, 6 clinical trials, a total of 2176 patients were included in the study. | 5 |
| Study characteristics | 18 | Four studies reported the use of steroids, and two reported the use of hyaluronidase. Four studies reported the use of peripheral vasodilators, including isoxsuprine and pentoxifylline. The use of antioxidants was also found to be very common: three studies reported the use of spirulina, eight studies reported the use of curcumin, and four studies reported the use of lycopene. In addition, other drugs such as omega 3, allicin, colchicine, and oxitard were used to treat OSF. Table 1 presents the results of all the included studies. The included studies were all published between 2011 and 2022 and mainly reported data from Asia including China and India. The minimum duration of the intervention was 1.5 months and the maximum duration was 10 months. The main outcomes were improvement in mouth opening, burning sensation, cheek flexibility, and tongue protrusion. | 5 |
| Risk of bias within studies | 19 | Not applicable | - |
| Results of individual studies | 20 | In 2012, Chole et al.(Chole et al., 2012) reviewed the literature on drug treatment for OSF to identify the role of various drugs in the treatment of OSF. With the emergence of new drugs for the treatment of OSF, we summarize the available pharmacological interventions for the treatment of OSF, describe the efficacy of contemporary and newly developed treatment modalities attempts to provide reference strategies for future research. Therefore, in this review, we collected relevant studies conducted in the past 10 years from 2012 to the present to systematically identify published randomized controlled trials (RCTs), non-RCT studies, and meta-analyses on various drugs for the treatment of OSF since December 2011 and update the literature with new clinical studies | 3 |
| Synthesis of results | 21 | Figure 1 shows the process of the literature search. The database search yielded a total of 270 studies, and the manual search yielded 9 studies. After eliminating duplicate studies, 117 studies were selected for further title and abstract screening. After screening, 68 studies were excluded as they did not meet the inclusion criteria. The remaining 49 studies were selected for full-text screening, and among the 49 studies, 35 met the criteria and were accepted. These 35 studies included 29 RCTs, 6 clinical trials, a total of 2176 patients were included in the study. Four studies reported the use of steroids, and two reported the use of hyaluronidase. Four studies reported the use of peripheral vasodilators, including isoxsuprine and pentoxifylline. The use of antioxidants was also found to be very common: three studies reported the use of spirulina, eight studies reported the use of curcumin, and four studies reported the use of lycopene. In addition, other drugs such as omega 3, allicin, colchicine, and oxitard were used to treat OSF. Table 1 presents the results of all the included studies. The included studies were all published between 2011 and 2022 and mainly reported data from Asia including China and India. The minimum duration of the intervention was 1.5 months and the maximum duration was 10 months. The main outcomes were improvement in mouth opening, burning sensation, cheek flexibility, and tongue protrusion. The results shows that drugs with evidence high quality were SMI combined with triamcinolone acetonide, lycopene, pentoxifylline, curcumin, and aloe vera, and those with evidence moderate quality were allicin, colchicine, omega 3, and oxitard. | 5-6 |
| Risk of bias across studies | 22 | Not applicable | - |
| Additional analysis | 23 | Not applicable | - |
| **DISCUSSION** | | |  |
| Summary of evidence | 24 | In conclusion, our findings found that steroids, hyaluronidase, pentoxifylline, antioxidants, omega 3, colicine, and allicin can alleviate the symptoms of OSF, for long-term treatment, lycopene is effective and has few side effects. Aloe vera is the most effective for relieving the symptoms of severe burning. Regardless of the number of treatment options available, abstaining from betel nut chewing is the best strategy to prevent OSF. Our review is intended only as a reference for clinical medication management, we hope that more high-quality meta-analyses, systematic reviews and multicenter RCTs with larger samples will provide more reference suggestions for the treatment of OSF in the future.  In a network meta-analysis comparing the efficacy of different treatment interventions for OSF(Gopinath et al., 2022), most interventions were found to be superior to placebo in improving clinical symptoms, such as mouth opening and burning sensation. Oxitard is superior to other interventions in improving mouth opening, aloe vera is superior in relieving burning sensation, and lycopene has the lowest propensity for side effects and can be considered the best safety agent. | 13 |
| Limitations | 25 | This review had a few limitations, the first of which was the small sample size; only one clinical trial involved more than 150 people. Second, patients could only be verbally advised not to chew betel nuts during the trial. However, there was no effective means to monitor and confirm that all patients have eliminated the harmful habits and thus the results might be biased to some extent. | 13 |
| Conclusions | 26 | In conclusion, our findings found that steroids, hyaluronidase, pentoxifylline, antioxidants, omega 3, colicine, and allicin can alleviate the symptoms of OSF, for long-term treatment, lycopene is effective and has few side effects. Aloe vera is the most effective for relieving the symptoms of severe burning. Regardless of the number of treatment options available, abstaining from betel nut chewing is the best strategy to prevent OSF. Our review is intended only as a reference for clinical medication management, we hope that more high-quality meta-analyses, systematic reviews and multicenter RCTs with larger samples will provide more reference suggestions for the treatment of OSF in the future. | 13 |
| **FUNDING** | | |  |
| Funding | 27 | Our study was supported by Natural Science Foundation of Hunan Province (No. 2021JJ70062); The Scientific Research Project of Hunan Health Commission (No. D202313048136); Chinese medicine research Project of Hunan Province (No. B2023048); Joint Fund Project of Hunan University of Chinese Medicine (No.2022XYLH120 and 2022XYLH134). | 14 |

*From:*  Moher D, Liberati A, Tetzlaff J, Altman DG, The PRISMA Group (2009). Preferred Reporting Items for Systematic Reviews and Meta-Analyses: The PRISMA Statement. PLoS Med 6(7): e1000097. doi:10.1371/journal.pmed1000097

For more information, visit: **www.prisma-statement.org**.

Page 2 of 2
